# SATB1 and SATB2 play opposing roles in c-Myc expression and progression of colorectal cancer

**DOI:** 10.18632/oncotarget.6651

**Published:** 2015-12-18

**Authors:** Mohammed A. Mansour, Toshinori Hyodo, Khondker Ayesha Akter, Toshio Kokuryo, Keisuke Uehara, Masato Nagino, Takeshi Senga

**Affiliations:** ^1^ Division of Cancer Biology, Nagoya University Graduate School of Medicine, Showa, Nagoya, 466-8550 Japan; ^2^ Biochemistry Section, Department of Chemistry, Faculty of Science, Tanta University, Tanta 31527, Egypt; ^3^ Department of Surgical Oncology, Nagoya University Graduate School of Medicine, Showa, Nagoya, 466-8550 Japan

**Keywords:** colorectal cancer, SATB2, c-Myc, SATB1

## Abstract

Special AT-rich sequence-binding protein 1 and 2 (SATB1/2) are nuclear matrix-associated proteins involved in chromatin remodeling and regulation of gene expression. SATB2 acts as a tumor suppressor in laryngeal squamous cell carcinoma and colon cancer, whereas SATB1 promotes the progression of numerous types of cancers. In this study, we examined the effects of SATB1 and SATB2 on the malignant characteristics of colorectal cancer cells. SATB1 and SATB2 expression were negatively correlated in colorectal cancer specimens. SATB1 expression was increased, whereas SATB2 expression was reduced, in colorectal cancer tissues compared to control tissues. Exogenous expression of SATB2 in colorectal cancer cells suppressed cell proliferation, colony formation and tumor proliferation in mice. c-Myc was reduced by SATB2 expression, and exogenous expression of c-Myc in SATB2-expressing cells restored proliferation, colony formation and *in vivo* tumor growth of colorectal cancer cells. We also showed that c-Myc reduction by SATB2 was mediated by the inactivation of ERK5. In contrast, SATB1 promoted c-Myc expression. The expression of SATB1 in colorectal cancer tissues was positively correlated with c-Myc expression, and SATB1 knockdown reduced c-Myc expression in colorectal cancer cells. Finally, we showed that SATB1 knockdown in colorectal cancer cells suppressed cell proliferation, colony formation and cell invasion. Our results reveal interesting features of how the structural homologs SATB1 and SATB2 exert opposing functions in colorectal tumorigenesis.

## INTRODUCTION

Colorectal cancer (CRC) is a major health problem worldwide due to its high prevalence and high mortality rates. Annually, more than 1 million new cases are clinically diagnosed, and more than 500,000 patients die from CRC each year. When diagnosed early, CRC is one of the most curable types of cancer, with a 90% cure rate [1]. Studies show that the majority of CRCs could be prevented by applying existing cancer prevention knowledge and by increasing the use of established screening examinations. Despite these advances, many patients with late stage and metastatic tumors will still succumb to the disease [2]. Therefore, additional diagnosis and treatment advances are required to combat the disease.

Special AT-rich sequence-binding protein 1 and 2 (SATB1/2) are nuclear matrix-associated proteins that are important for growth and developmental processes [3,4]. SATB2 has been identified as a putative cleft palate gene and was first named as a homolog of the DNA matrix binding protein SATB1. SATB1/2 are highly conserved across vertebrates from zebrafish to chickens to mammals [5]. SATB2 expression is tissue specific, and the glandular cells lining the lower gastrointestinal (GI) tract express high levels of this protein [3]. Nevertheless, the normal colorectal mucosa contains very low to undetectable levels of SATB1 [6]. These proteins fold chromatin into loops and recruit chromatin remodeling and modifying proteins to these DNA loops to either activate or repress gene transcription [7–11]. SATB1 overexpression was observed in many types of human tumors, where it promotes cancer cell growth and metastasis by altering the gene expression profile [12–23]. SATB1 activates many genes that have roles in carcinogenesis, including ABL1, ERRB2, MMP2, E-CADHERIN, VEGFB, KISS1 and TGFB1 [7,24,25]. SATB1 expression in most cancers including CRC is associated with progression, poor prognosis and microsatellite instability (MSI), and SATB1 expression correlates with the loss of SATB2 expression [26].

SATB2 has been increasingly linked to colorectal [27] and head and neck [28] cancers. The effects of SATB2 expression on metastasis and prognosis of CRC are different from those on other types of cancer [29]. In CRC, SATB2 expression is correlated with a favorable prognosis, less MSI and enhancement of the benefits of adjuvant therapy [27]. These studies indicate a possible negative correlation between the structural homologs SATB1 and SATB2 in cancer; however, a detailed analysis of how the two proteins exert opposing functions in tumorigenesis has not yet been carried out. In this report, we examined the functions of SATB1 and SATB2 in CRC. We showed that SATB1 and SATB2 are negatively correlated in CRC and that they have different effects on colorectal tumorigenesis and expression of c-Myc. While SATB2 inhibits c-Myc expression via inactivation of MEK5/ERK5 signaling, SATB1 activates the transcription of c-Myc.

## RESULTS

### Ectopic expression of SATB2 inhibits c-Myc expression

**Figure 1:**
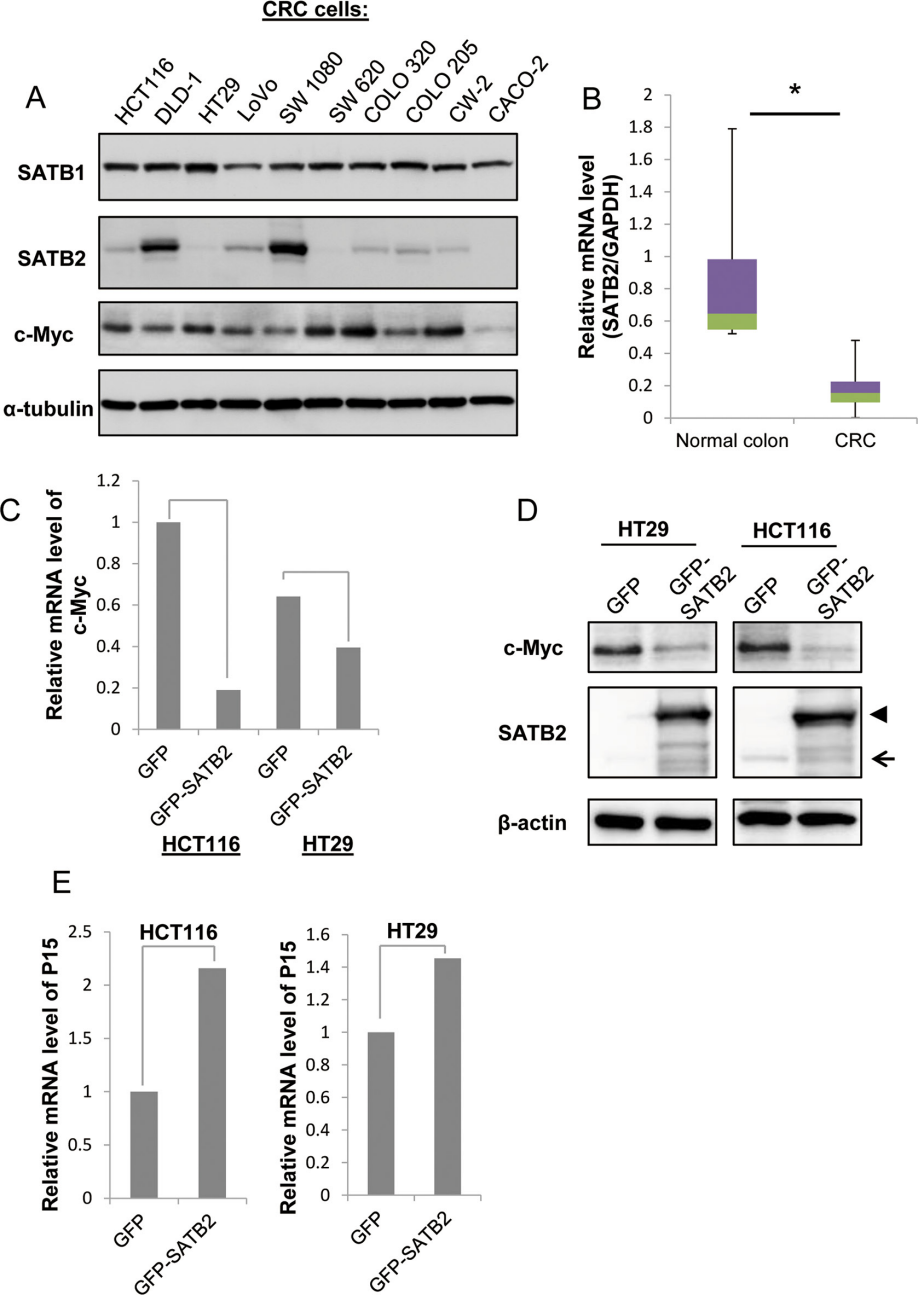
Ectopic expression of SATB2 inhibits c-Myc expression **A.** The expression levels of SATB1, SATB2 and c-Myc were examined by immunoblotting. **B.** The level of SATB2 mRNA in colorectal cancer specimens and normal colorectal tissues was evaluated by quantitative RT-PCR. The graph indicates the relative SATB2 mRNA level. **C.** The level of c-Myc mRNA in HCT116 and HT29 cells expressing either GFP or GFP-SATB2 was evaluated by quantitative RT-PCR. The graph indicates the relative c-Myc mRNA level. **D.** HCT116 and HT29 cells constitutively expressing GFP (GFP) or GFP-tagged SATB2 (GFP-SATB2) were generated by retroviral infection. The expression levels of c-Myc or SATB2 proteins in each cell line were examined by immunoblotting. The arrow indicates endogenous SATB2 and the arrowhead indicates GFP-SATB2. **E.** The levels of P15 mRNA in HCT116 and HT29 cells expressing either GFP or GFP-SATB2 were evaluated by quantitative RT-PCR. The graph indicates the relative P15 mRNA level.

To investigate the functions of the SATB1 and SATB2 proteins in colorectal tumorigenesis, we determined the expression of the two proteins in CRC cells by immunoblotting. As shown in Figure 1A, SATB1 was highly expressed in all CRC cells studied; however, SATB2 exhibited very low to undetectable levels in all CRC cells except DLD-1 and SW1080 cells. Furthermore, the expression of the well-known oncogene c-Myc was consistent with the protein expression of SATB1 but not SATB2. Then, we examined SATB2 mRNA levels in human colorectal cancer tissues using qRT-PCR analysis. SATB2 mRNA levels were significantly reduced in human colorectal cancer specimens (*n* = 42) compared to the normal controls (*n* = 11) (Figure 1B). We next examined the effects of the exogenous expression of SATB2 on colorectal cancer cells. We established that HCT116 and HT29 cells constitutively expressed GFP or GFP-SATB2 by retrovirus infection. The level of exogenously expressed GFP-SATB2 was significantly higher than that of endogenous SATB2 (Figure 1D).

The well-known oncogene c-Myc is activated by several effector molecules, including ERK5 [30]. Recently, we reported that SATB2 inactivates ERK5 signaling to suppress the progression of colorectal cancer[31]. Therefore, we investigated the expression of c-Myc in response to SATB2 expression. The expression of GFP-SATB2 repressed the expression of c-Myc at both the mRNA level (Figure 1C) and the protein level (Figure 1D). c-Myc serves as a repressor of cyclin-dependent kinase (CDK) inhibitors such as P15 (a cell growth regulator that controls cell cycle G1 progression) through the interaction of the c-Myc-Max heterodimer with transcription factors such as MIZ-1 [32]. Consistent with SATB2-mediated c-Myc suppression, the CDK inhibitor P15 was upregulated by SATB2 expression in HCT116 and HT29 cells (Figure 1E).

### Expression of c-Myc restores the malignant characteristics of SATB2-expressing cells

**Figure 2:**
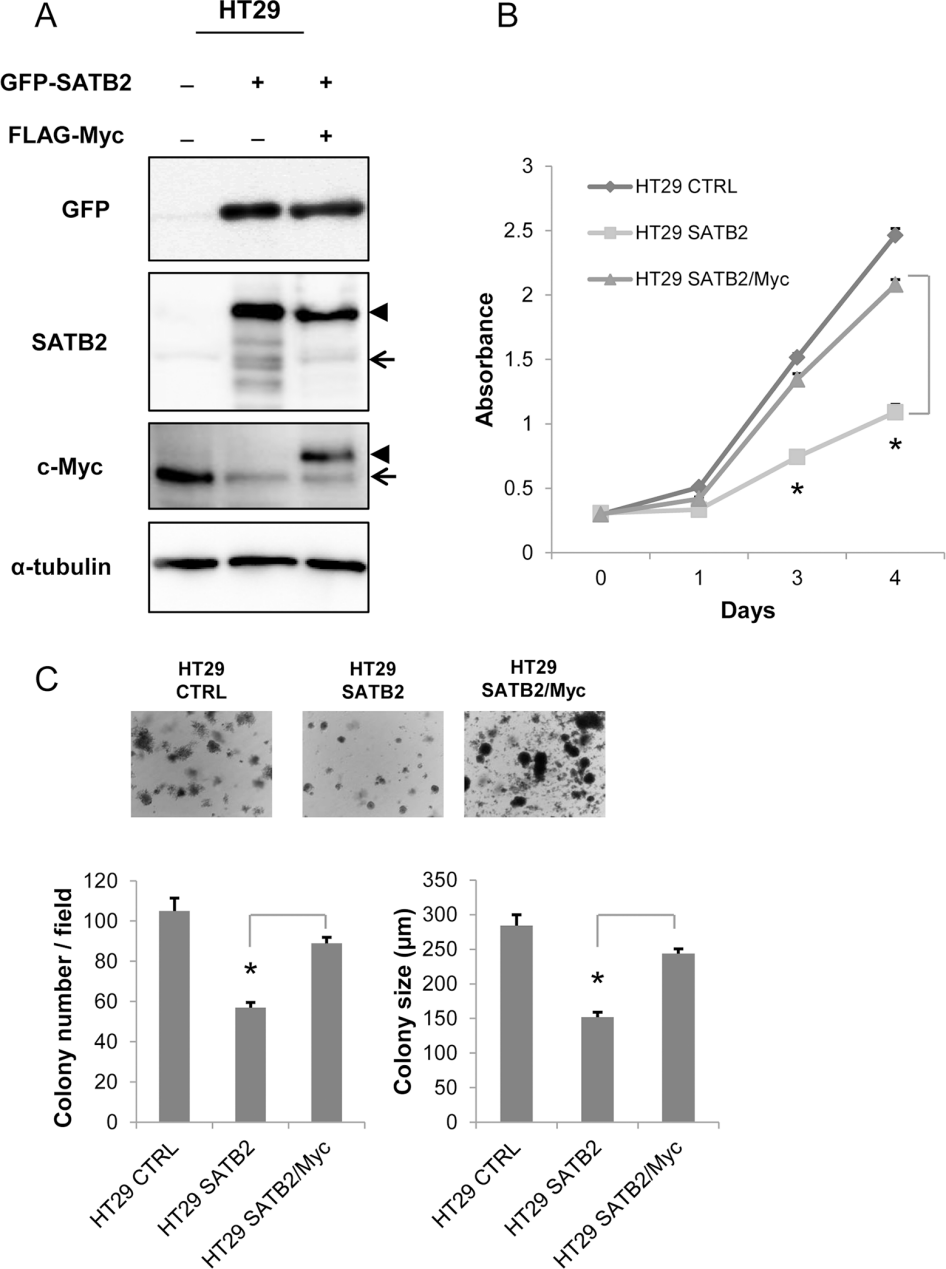
Ectopic expression of c-Myc restores the proliferation of SATB2-expressing cells **A.** HT29 cells constitutively expressing GFP-SATB2 or GFP-SATB2 and Flag-Myc were established by retrovirus infection. The expression of the indicated proteins in the cell lines were examined by immunoblotting. The arrow and the arrowhead indicate the endogenous protein and the exogenous protein, respectively. **B.** The numbers of viable cells at the indicated time points were determined using a Cell Counting Kit-8 assay for HT29 cells. **C.** CTRL, SATB2 and SATB2/Myc HT29 cells were subjected to a colony formation assay. Representative images are shown for HT29 cells, and the graphs indicate the average number and size of colonies per field (*P < 0.05).

We developed HT29 cells expressing either SATB2 alone (SATB2) or both SATB2 and c-Myc (SATB2/Myc) by retroviral infection. The endogenous level of c-Myc in SATB2 or SATB2/Myc cells was clearly reduced; however, exogenous c-Myc (Flag-Myc) in SATB2/Myc cells was expressed at a level similar to that of the endogenous protein in the parental cells (Figure 2A). To explore whether c-Myc expression could restore the proliferation of SATB2-expressing cells, a cell proliferation assay was performed. As shown in Figure 2B, c-Myc expression significantly restored the proliferation of SATB2-expressing cells. We also examined the anchorage-independent growth of SATB2 and SATB2/Myc cells by culturing the cells in soft agar for two weeks. As shown in Figure 2C, SATB2/Myc cells formed more colonies and larger colonies than SATB2 cells (Figure 2C).

**Figure 3:**
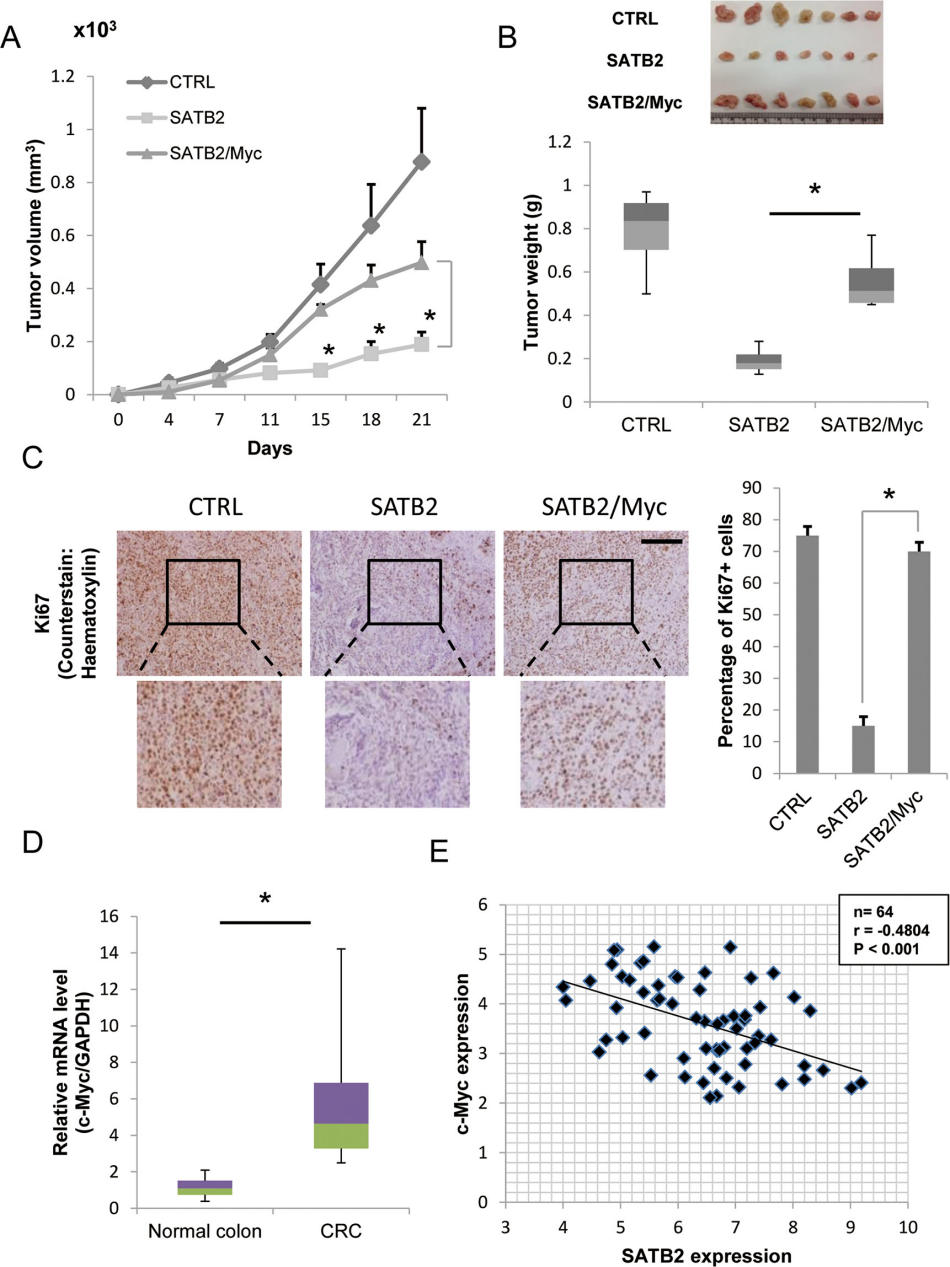
Ectopic expression of c-Myc restores *in vivo* tumor growth of SATB2-expressing cells **A.** CTRL, SATB2 and SATB2/Myc HT29 cells were subcutaneously injected into the femurs of nude mice, and tumor volumes were measured. The graph shows the average volume of seven tumors corresponding to each cell line (*P < 0.05). **B.** Twenty-one days after tumor injection, the mice were sacrificed and the tumor weights were measured. The picture shows the extracted tumors, and the graph indicates the average tumor weight of the seven tumors derived from each cell line (*P < 0.05). **C.** Ki67 immunohistochemistry of xenograft tumors extracted from CTRL, SATB2 or SATB2/Myc-injected nude mice. Expression of Ki67 (positive cells are brown) was visualized using a light microscope (scale bar = 50 μm). The percentage of positive cells in the different tumors was quantified (right). **D.** The levels of c-Myc mRNA in colorectal cancer specimens and normal colorectal tissues were evaluated by quantitative RT-PCR. The graph indicates the relative c-Myc mRNA level. **E.** The relative expression levels of SATB2 and c-Myc mRNA normalized to glyceraldehyde-3-phosphate dehydrogenase (GAPDH) mRNA in 64 clinical tissues were determined by quantitative RT-PCR. The Pearson correlation coefficient (r) is shown.

Studies of *in vivo* xenograft tumors also confirmed the tumor-promoting role of c-Myc expression in SATB2-expressing cells. The tumor volume and weight of SATB2 cells were significantly reduced compared with that of the control (CTRL) cells (Figure 3A & 3B). Ectopic expression of c-Myc in SATB2 cells (SATB2/Myc) restored the tumor volume and weight compared with that of SATB2 cells (Figure 3A & 3B). To confirm the increased proliferative ability of the cells, tumors from orthotopically-injected mice were removed and processed for further histological and immunohistochemical analysis. As shown in Figure 3C, tumors from the CTRL and the SATB2/Myc groups exhibited increased cell proliferation as indicated by the strong staining of Ki67 compared with that of the SATB2 group. These results indicate that expression of c-Myc in SATB2-expressing cells led to increased tumor growth and proliferative ability of the cells. In the clinical arm of the study, we measured the expression of c-Myc in colorectal cancer specimens (*n* = 32) and normal colon samples (*n* = 40). Consistent with the previous results, c-Myc expression was significantly increased in the colorectal cancer tissues compared to the normal colon samples (Figure 3D). Then, we analyzed the expression of both SATB2 and c-Myc in the clinical samples (*n* = 64) using Pearson correlation analysis. SATB2 expression was significantly negatively correlated with c-Myc expression (Figure 3E), confirming the negative association between the two proteins in the *in vitro* studies.

### SATB2 inhibits c-Myc expression via an ERK5-dependent mechanism

**Figure 4:**
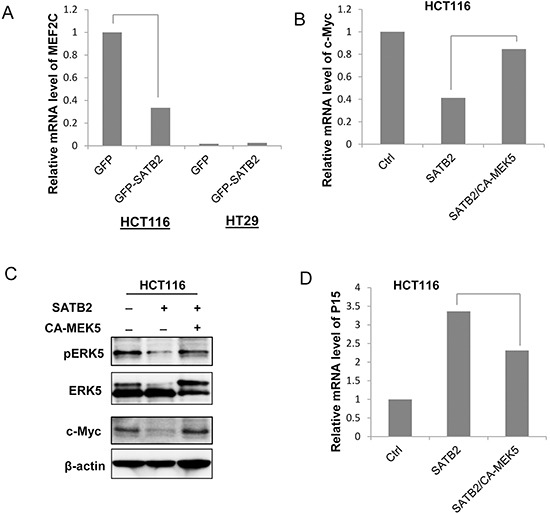
SATB2 inhibits c-Myc expression via an ERK5-dependent mechanism **A.** The MEF2C mRNA expression level in GFP and GFP-SATB2-expressing cells was analyzed by quantitative RT-PCR analysis. The graph indicates the relative MEF2C mRNA level. **B.** The c-Myc mRNA expression levels in Ctrl, SATB2 and SATB2/CA-MEK5 cells were analyzed by quantitative RT-PCR analysis. The graph indicates the relative c-Myc mRNA level. **C.** The expression levels of pERK5, ERK5, c-Myc and β-actin were examined by immunoblotting. **D.** The P15 mRNA expression levels in Ctrl, SATB2 and SATB2/CA-MEK5 cells were analyzed by quantitative RT-PCR analysis. The graph indicates the relative P15 mRNA level.

As mentioned earlier, our recent studies reported a tumor-suppressing function of SATB2 in CRC via the inactivation of MEK5/ERK5 signaling [31,33]. Among ERK5 substrates, ectopic expression of SATB2 inhibited the expression of c-Myc (Figure 1C & 1D) and MEF2C (Figure 4A) in HCT116 and HT29 cells. Meanwhile, the activation of ERK5 by the expression of constitutively active MEK5 (CA-MEK5) in SATB2-expressing cells restored the mRNA and protein expression levels of c-Myc (Figure 4B & 4C). As a result, the CDK inhibitor P15 was upregulated by SATB2 expression and downregulated by the activation of ERK5 (Figure 4D).

### SATB1 is upregulated in colon cancer samples, and silencing of SATB1 inhibits cell proliferation *in vitro*

**Figure 5:**
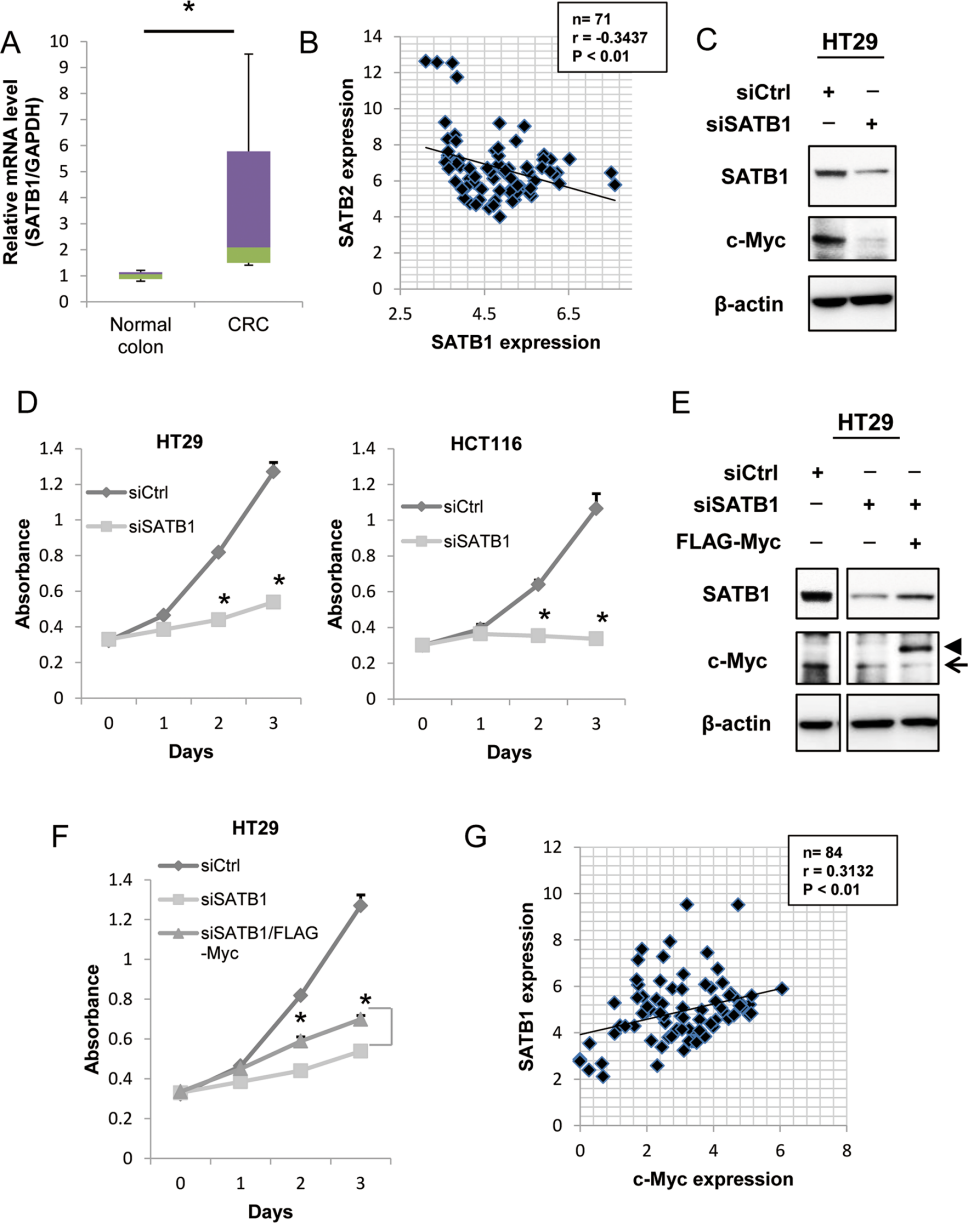
Depletion of SATB1 inhibits cancer cell proliferation *in vitro* **A.** The levels of SATB1 mRNA in colorectal cancer specimens and normal colorectal tissues were evaluated by quantitative RT-PCR. The graph indicates the relative SATB1 mRNA level. **B.** The relative expression levels of SATB1 and SATB2 mRNA normalized to glyceraldehyde-3-phosphate dehydrogenase (GAPDH) mRNA in 72 clinical tissues were determined by quantitative RT-PCR. The Pearson correlation coefficient (r) is shown. **C.** The expression levels of SATB1, c-Myc and β-actin were examined by immunoblotting. **D.** The number of viable cells at the indicated time points was determined using a Cell Counting Kit-8 assay for HT29 and HCT116 cells. **E.** HT29 cells constitutively expressing Flag-Myc were established by retrovirus infection. Cells were transfected with control or SATB1 siRNA, and the expression levels of the indicated proteins were examined by immunoblotting. The arrow and the arrowhead indicate the endogenous protein and the exogenous protein, respectively. **F.** The number of viable cells at the indicated time points was determined using a Cell Counting Kit-8 assay for HT29 cells. **G.** The relative expression levels of SATB1 and c-Myc mRNA normalized to glyceraldehyde-3-phosphate dehydrogenase (GAPDH) mRNA in 84 clinical tissues were determined by quantitative RT-PCR. The Pearson correlation coefficient (r) is shown.

To investigate the function of SATB1 in colorectal cancer, we first examined SATB1 mRNA levels in human colorectal cancer tissues by qRT-PCR. SATB1 mRNA levels were increased in the human colorectal cancer specimens (*n* = 20) compared to the normal controls (*n* = 9) (Figure 5A). Then, we analyzed the expression of both SATB1 and SATB2 in clinical samples (*n* = 71) using Pearson correlation analysis. SATB1 expression was significantly negatively correlated with SATB2 expression (Figure 5B). Therefore, the two proteins might have opposing functions in CRC progression.

We next examined the effects of the transient knockdown of SATB1 on colorectal cancer cells using short interfering RNA (siRNA) transfection. Transfection of SATB1 siRNA significantly reduced the SATB1 expression in HT29 cells (Figure 5C). Importantly, knockdown of SATB1 inhibited the expression of c-Myc (Figure 5C). To determine whether SATB1 suppression had any effect on cell growth, we performed a cell proliferation assay. As shown in Figure 5D, the knockdown of SATB1 significantly reduced the proliferation of HT29 and HCT116 cells *in vitro*. We tested whether exogenous expression of c-Myc could restore the proliferation of SATB1-knockdown cells. HT29 cells that constitutively expressed Flag-Myc were generated. The cells were depleted of SATB1 by siRNA transfection, and the levels of endogenous c-Myc and Flag-Myc were examined by immunoblotting. As shown in Figure 5E, SATB1 depletion reduced the level of endogenous c-Myc, but not Flag-Myc. A cell proliferation assay demonstrated that exogenous expression of Flag-Myc clearly restored the proliferation of SATB1-knockdown cells (Figure 5F). Finally, we examined the expression levels of SATB1 and c-Myc in colorectal cancer tissues. The expression of SATB1 in clinical samples (*n* = 84) was significantly positively correlated with c-Myc expression (Figure 5G).

### SATB1 knockdown inhibits colony formation, cell migration and invasion

**Figure 6:**
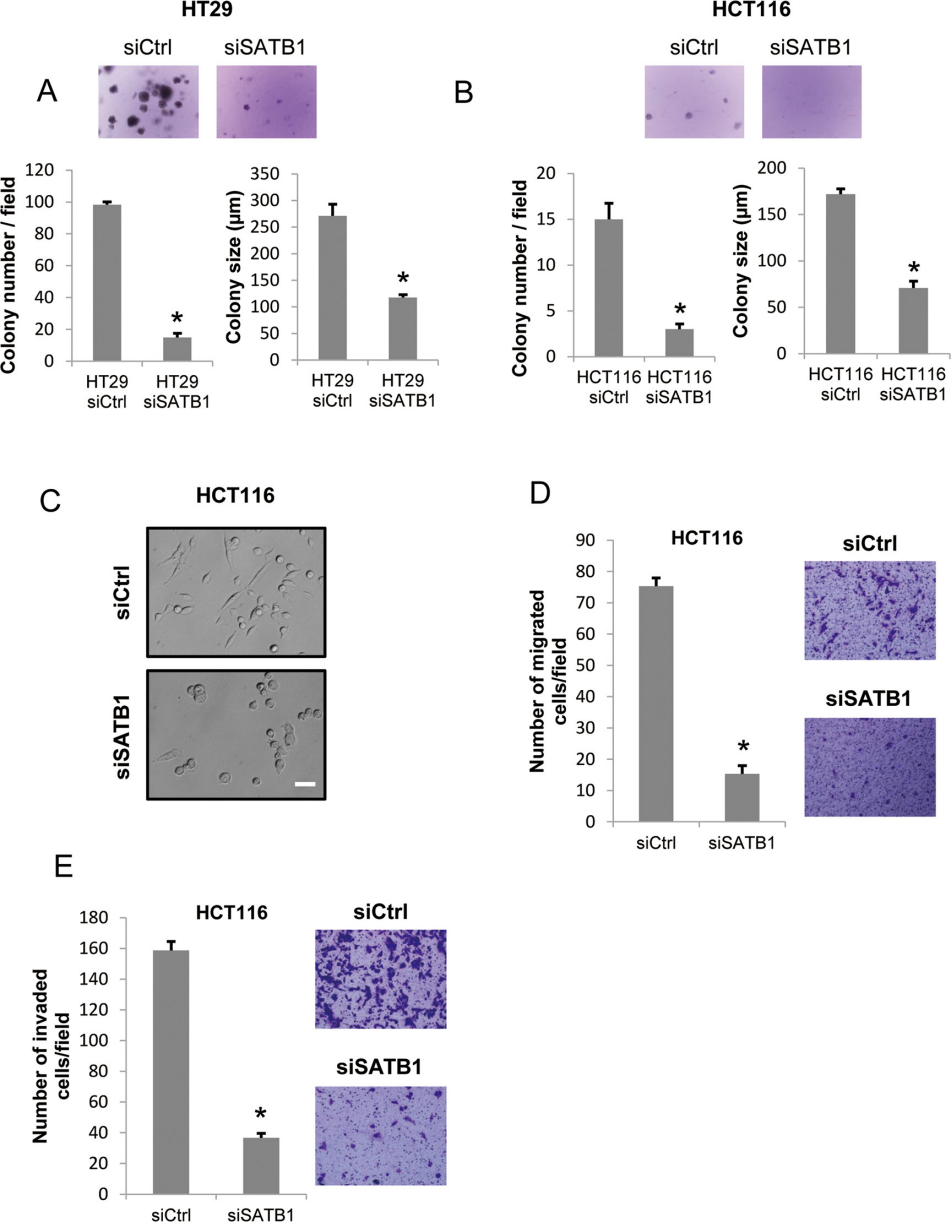
Depletion of SATB1 represses colony formation, cell migration and invasion **A.** Control or SATB1 siRNA-transfected HT29 cells were subjected to a colony formation assay. Representative images are shown, and the graphs indicate the average number and size of colonies per field (*P < 0.05). **B.** siRNA-transfected HCT116 cells were subjected to a colony formation assay. Representative images are shown, and the graphs indicate the average number and size of colonies per field (*P < 0.05). **C.** The pictures are representative images showing the cellular morphology of the siRNA-transfected HCT116 cells (Scale bar = 50 μm). **D.** siRNA-transfected HCT116 cells were subjected to the migration assay. Representative images of migrated cells are shown, and the graph indicates the average number of migrated cells per field (*P < 0.05). **E.** siRNA-transfected HCT116 cells were subjected to an invasion assay. Representative images of invaded cells are shown, and the graph indicates the average number of invaded cells per field (*P < 0.05).

To explore the function of SATB1 in other aspects of cancer cells, we analyzed anchorage-independent growth (colony formation), cell migration and invasion. We first tested whether SATB1 knockdown inhibits the anchorage-independent growth of colorectal cancer cells. HT29 or HCT116 cells were transfected with siRNAs and cultured in soft agar. Two weeks later, the number of colonies and the average colony size were determined. The results showed a clear suppression of the growth of SATB1 siRNA-transfected cells in the absence of cell adhesion to the extracellular matrix (Figure 6A & 6B). We next examined the migration and invasion of SATB1 siRNA-transfected HCT116 cells that exhibited a round-shape morphology compared to the control siRNA-transfected cells (Figure 6C). We used a modified Boyden chamber assay. The cells (serum-starved) were placed on the upper surface of a filter coated with fibronectin and then allowed to migrate to the bottom surface filled with serum-containing medium. We counted the cells that migrated to the bottom surface 24 h after the seeding. The migration of SATB1 siRNA-transfected HCT116 cells was clearly suppressed compared with that of the Ctrl (control) siRNA-transfected cells (Figure 6D). We next examined cell invasion using Matrigel-coated Boyden chambers. We seeded either control or SATB1 siRNA-transfected HCT116 cells in the upper chamber (serum-free) and allowed the cells to invade through a Matrigel-coated filter toward the serum-containing medium. After 24 h, the cells were fixed and the number of invaded cells was counted. As shown in Figure 6E, the invasion of SATB1 siRNA-transfected HCT116 cells was clearly suppressed compared to that of the control siRNA-transfected cells.

**Figure 7:**
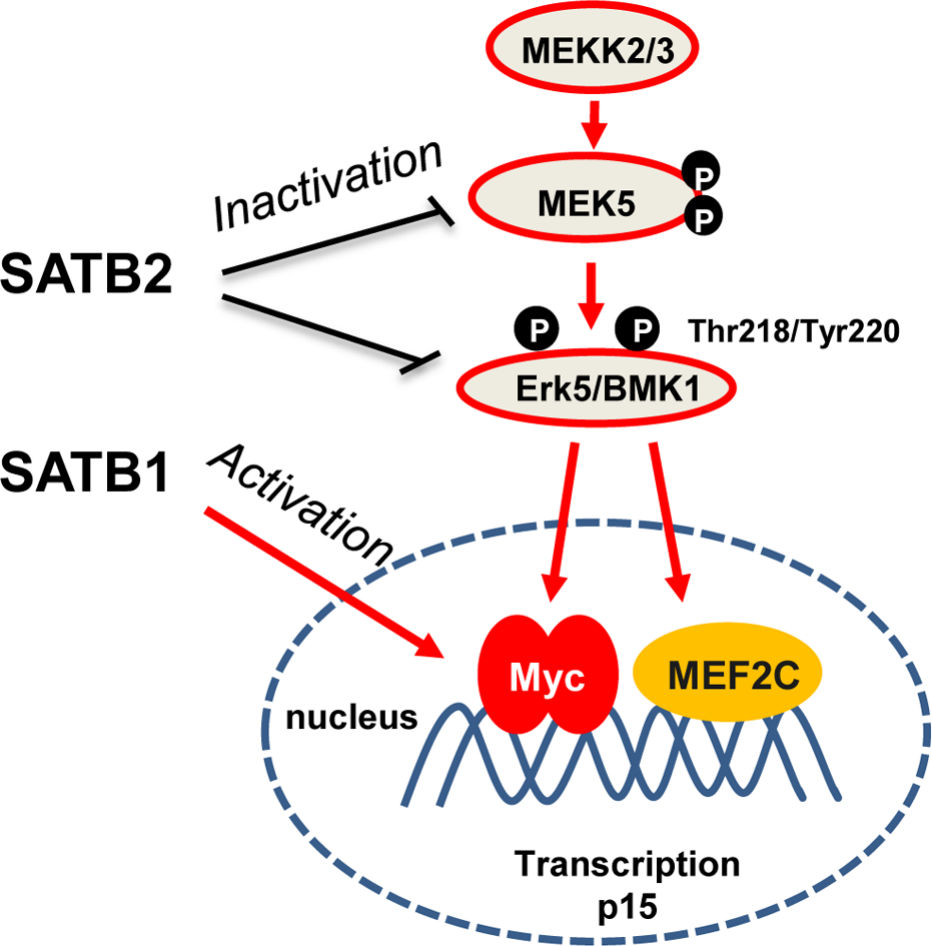
SATB1 and 2 have opposing roles in colorectal tumorigenesis and Myc expression SATB2 regulates the transcription of several genes to inactivate ERK5 signaling, which in turn represses the expression of c-Myc and MEF2C. However, SATB1 promotes the expression of Myc most likely by binding to the promoter region.

Collectively, SATB1 and SATB2 exhibit opposing roles in colorectal tumorigenesis and c-Myc expression. c-Myc reduction by SATB2 was mediated by the inactivation of MEK5/ERK5 signaling. However, SATB1 promotes cancer cell proliferation, anchorage-independent growth, migration and invasion in a manner that is dependent on the induction of c-Myc (Figure 7).

## DISCUSSION

In this report, we examined the effects of SATB2 expression and SATB1 knockdown in different colorectal cancer cell lines. The proliferation and anchorage-independent growth of these cell lines were significantly repressed by SATB2 expression and by silencing of SATB1. The results of the current study showing a tumor-suppressing function of SATB2 in HT29 cells are consistent with our recent reports concerning the function of SATB2[31,33]. Accumulating evidence has shown that the chromatin modifier SATB1 is associated with malignancies [12–24]. Together with these previous findings, our results clearly indicate that SATB1 promotes cancer cell growth and invasion by altering the gene expression profile. In parallel, a significant negative correlation of SATB1 and SATB2 expression was observed in the clinical samples. Alignment of the SATB1 and SATB2 amino acid sequences showed an identity of 61% and a similarity of 73% [3]. However, the two proteins have been reported to have antagonistic functions in embryonic stem cell differentiation [34].

The transcription factor c-Myc is a member of the Myc gene family, which mediates various physiological functions including cell cycle control, apoptosis, protein synthesis, and cell adhesion [35,36]. The c-Myc gene is activated in cancer cells by chromosomal translocation, rearrangement, and amplification. [36]. c-Myc also plays a crucial role in malignant transformation. Overexpression of c-Myc is associated with the features of many human malignancies, such as proliferation, invasion, angiogenesis, and metastasis [36,37]. We showed that c-Myc expression was critical for SATB1/2-mediated tumor progression or tumor suppression. Our results suggest that c-Myc may be a potential therapeutic target for colorectal cancers with high expression of SATB1 or low expression of SATB2.

In this study, we showed that SATB1 and its homolog SATB2 mediate opposing functions in the regulation of colorectal carcinogenesis and c-Myc expression. Recently, we reported that SATB2 inactivates MEK5/ERK5 signaling to suppress colorectal cancer progression [31]. ERK5 is activated by active phosphorylation of the Thr and Tyr residues within the T–E–Y motif in the activation loop of the kinase domain. Then, the activated ERK5 autophosphorylates its C-terminal residues including Ser769/773/775 [38]. The downstream substrates of ERK5 include MEF2C, c-Fos, Fra 1, Sap-1, c-Myc and NF-κB, most of which are oncogenes [30,35,39,40]]. In this study, both c-Myc and MEF2C were inhibited by SATB2 expression. Meanwhile, the activation of ERK5 in SATB2-expressing cells could restore c-Myc expression. Therefore, we assume that SATB2 inhibits the expression of c-Myc in CRC cells via an ERK5-dependent mechanism.

SATB1 was originally identified as a chromatin remodeling protein that binds to gene promoters to regulate gene and protein expression levels [24–26]. SATB1 overexpression was observed in many types of human tumors, where it promotes cancer cell growth and metastasis by altering the gene expression profile [13–20]. Here, we showed that the expression of SATB1 in colorectal cancer tissues was positively correlated with c-Myc expression and that SATB1 knockdown reduced c-Myc expression in colorectal cancer cells. Cai et al. [42] showed that SATB1 binds to a specific promoter sequence from the transcription start site of the Myc gene. A similar SATB1 binding sequence is found in the human and mouse Myc promoter region [41,42]. Recently, Chen et al. [43] identified Myc as a downstream target of SATB1 that promotes pancreatic cancer progression depending on Myc activation. Because SATB1 is a chromatin modifier that regulates gene expression by binding to the 5′ base-unpairing regions (BURs) [7], future studies could uncover the molecular mechanisms of c-Myc activation by SATB1 in colorectal cancer.

To the best of our knowledge, these are the first results to clearly demonstrate that SATB1 and SATB2 exert opposing functions in CRC proliferation, anchorage-independent growth and invasiveness, and in regulating c-Myc expression via two different mechanisms. The results elucidate the importance of SATB1 and SATB2 in understanding oncogenic c-Myc-induced colorectal tumorigenesis, making SATB1 a promising therapeutic target for colorectal cancer.

## MATERIALS AND METHODS

### Cells and antibodies

HCT116 and HT29 cells were obtained from ATCC and cultured in DMEM supplemented with 10% FBS and antibiotics. Cells were authenticated by short tandem repeat analysis using the GenePrint® 10 System (Promega, Madison, WI) in 2014. The HEK293T cells for retrovirus production were maintained in DMEM with 10% FBS. Antibodies were obtained from the following companies: anti-SATB1 and anti-ERK5 antibodies, Cell Signaling (Danvers, MA, USA); anti-Myc antibody (9E10A) and anti-SATB2 antibody, Abcam (Cambridge, UK); anti-GFP antibody, Neuro Mab (Davis, CA); anti-phospho-ERK5 antibody (Thr218/Tyr220), Affinity BioReagents (Golden, CO); and anti-α-tubulin and anti-β-actin antibodies, Sigma-Aldrich (St. Louis, MO).

### Generation of stable cell lines

Full-length SATB2 was PCR amplified from a cDNA library of HCT116 cells. SATB2 was cloned into the pQCXIP vector with an N-terminal GFP tag. c-Myc cDNA was cloned into PQCXIP vector with an N-terminal FLAG tag. Then, the plasmids were transfected into 293T cells together with the pVPack-GP and pVPack-Ampho vectors using Lipofectamine 2000 (Invitrogen, Carlsbad, CA). Forty-eight hours after transfection, the supernatants were added to the cells along with 2 μg/ml polybrene (Sigma-Aldrich), and the infected cells were selected by incubating with 1 μg/ml puromycin for 2 days. The constitutively active form of MEK5 was generated by substituting Ser311 and Thr315 with aspartic acid by PCR; the mutant was cloned into the pQCXIP vector with a Flag tag at the N-terminus. To establish a cell line constitutively expressing the active form of MEK5 and SATB2, recombinant retrovirus that encoded GFP-SATB2 and active MEK5 were infected into HCT116 cells and selected with puromycin and neomycin.

### Western blotting

Cell lysates were loaded on SDS-polyacrylamide gels for electrophoresis and transferred to polyvinylidene difluoride membranes (Millipore). The membranes were blocked with 1% skim milk for 1 h and then incubated with primary antibodies for 1 h. The membranes were then washed with TBS-T for 15 min and incubated with HRP-labeled secondary antibodies. The signals were detected using the ECL system (GE Healthcare BioSciences). The signal intensities were measured using Light Capture II equipped with a CS analyzer (ATTO Corp., Tokyo, Japan).

### Patients and ethics statement

Colorectal cancer samples and normal colorectal tissues were obtained from patients who underwent surgery at the Nagoya University Hospital in 2012. For the detection of SATB1, SATB2 and c-Myc expression by qRT-PCR, normal colon tissues and CRC tissues (stage II– IV) were collected from male and female patients (Age (yr): 30-60). The study was approved by the institutional review board of the Nagoya University Hospital and conformed to the standards set by the Declaration of Helsinki. All participants provided written informed consent to participate in the study.

### Quantitative PCR analysis

RNA was extracted from colorectal cancer samples and cells using the RNeasy Mini Kit (Qiagen, Venlo, Netherlands), and cDNA was generated using PrimeScript Reverse Transcriptase (TAKARA, Tokyo, Japan). The colorectal cancer samples were obtained from patients at the Nagoya University Hospital with informed consent. PCR was performed using the SYBR Premix Ex Taq™ II (TAKARA), and the Thermal Cycler Dice™ Real Time System TP800 (TAKARA) was used for the analysis. The relative mRNA expression levels were normalized to GAPDH. The sequences of primers used to amplify each gene were 5′-AGGTGGAGGAGTGGGTGTCGCTGTT-3′ and 5′-CCGGGAAACTGTGGCGTGATGG-3′ (GAPDH), 5′-AGCCCAGCAGTCCTTAAACC-3′ and 5′-CAGTTCATCGCGTACCCACT-3′ (SATB1), 5′-CTTTGCAAGAGTGGCATTCA-3′ and 5′-GTTGTCGGTGTCGAGGTTTT-3′ (SATB2), 5′-CATCAGCACAACTACGCAGC-3′ and 5′-GCTGGTGCATTTTCGGTTGT-3′ (c-Myc), 5′-CGGGGACTAGTGGAGAAGGTG-3′ and 5′-CCATCATCATGACCTGGATCGC-3′ (P15), and 5′-CGAGATGCCAGTCTCCATCC-3′ and 5′-GTGAGCCAGTGGCAATAGGT-3′ (MEF2C).

### siRNA transfection

siRNAs were obtained from Sigma-Aldrich (St. Louis, MO, USA). The sequence of the siRNA used to knockdown SATB1 is 5′-AUAGGUGUUGAUACGAGCCCA-3′ (siSATB1). The sequence of the control siRNA that targets luciferase is 5′-CUUACGCUGAGUACUUCGATT-3′. The cells were transfected with 50 nM of the siRNA using Lipofectamine RNAiMAX (Invitrogen, Carlsbad, CA, USA) according to the manufacturer's instructions.

### Cell proliferation assay

Cells were cultured in 96-well plates, and the number of viable cells at the indicated time points were evaluated using the Cell Count Kit 8 assay (Dojindo, Tokyo, Japan).

### Colony formation assay

Cells (1 × 10^4^) were mixed with 0.36% agar in DMEM supplemented with 10% FBS and overlaid onto a 0.72% agarose layer in 6-well plates. After 2 weeks of incubation, the number and size of the colonies in five randomly selected fields were counted. Three independent experiments were performed, and the data are shown as the mean ± SE.

### Xenograft tumor assay

Male BALB/c Slc-nu/nu mice (5 to 6 weeks old) were purchased from Japan SLC (Hamamatsu, Japan). The study was performed in accordance with the guidelines issued by the Animal Center at the Nagoya University School of Medicine. Animals were housed in covered/filtered boxes under controlled temperature and humidity conditions in accordance with the Guidelines laid down by the NIH in the USA regarding the care and use of animals for experimental procedures. Animals were fed standard mouse chow and water ad libitum for an acclimation period of 1 week prior to the initiation of the study protocol. After acclimation, a total of 7.5 × 10^6^ HT29 cells (CTRL, SATB2 & SATB2/Myc) were suspended in 0.1 ml PBS and subcutaneously injected into both sides of the femoral area of the mice. Tumor growth and overall health of the mice were monitored twice per week. The tumor volumes were calculated using the formula l*w*h/2 (mm^3^). Mice who suffered unnecessarily, either acutely or chronically, at any stage of the experiment were excluded. At day 21 post-implantation, the mice were euthanized in a CO_2_ cage, and the tumors were extracted by standard surgery for the determination of tumor weight.

### Immunohistochemistry

Immunohistochemical staining was performed in HT29 tumors extracted from the nude mice. Paraffin-embedded sections (4 μm) were deparaffinized, re-hydrated and microwave-heated for 15 min in 0.01 mol l^−1^ citric acid buffer (pH 6.0) for antigen retrieval. Then, 3% hydrogen peroxide was applied to block endogenous peroxidase activity. After 15 min of blocking with normal serum (Invitrogen), the primary antibody was applied and the samples were incubated overnight at 4°C. The slides were washed three times with PBS for 5 min each. The slides were incubated with the biotinylated secondary antibody and the streptavidin–biotin complex, each for 30 min, and then washed three times at room temperature. After rinsing with PBS, the slides were immersed for 10 min in 3,3′-diaminobenzidine (Sigma) solution (0.4 mg ml^−1^, with 0.003% hydrogen peroxide) and monitored under the microscope. The reaction was stopped with distilled water, and the slides were counterstained with hematoxylin, dehydrated, and coverslipped. The primary antibody (Ki-67) was purchased from Novus Biologicals, USA.

### Migration assay

To measure cell migration using Boyden chambers, a filter (8 μm pore size, 6.5 mm membrane diameter) was pre-coated with fibronectin overnight, and 2 × 10^5^ cells were seeded onto the upper surface of the chamber with DMEM and 0.1% BSA (serum-free). However, the lower chamber was filled with DMEM and 10% FBS (chemo-attractant). Eighteen hours after seeding, the cells were fixed with 70% methanol and stained with 0.5% crystal violet. The cells that migrated through the lower surface of the filters were counted in five randomly selected fields. Three independent experiments were performed, and the data are shown as the mean ± SE.

### Invasion assay

To measure cell invasion using Boyden chambers, a filter (8 μm pore size, 6.5 mm membrane diameter) was pre-coated with Matrigel (a mixture of matrix molecules including laminin, collagen type IV and entactin) overnight, and 2 × 10^5^ cells were seeded onto the upper surface of the chamber with DMEM and 0.1% BSA (serum-free). The lower chamber was filled with DMEM and 10% FBS (chemo-attractant). Twenty-four hours after seeding, the cells were fixed with 70% methanol and stained with 0.5% crystal violet. The cells that invaded the lower surface of the filters were counted in five randomly selected fields. Three independent experiments were performed, and the data are shown as the mean ± SE.

### Statistical analysis

The statistical analysis is indicated in each corresponding figure legend. All data are represented as the mean ± SE from 3 independent assays. All analyses were examined using the SigmaPlot program version 10.0 (Systat Software, Inc., San Jose, CA). *P* values were calculated from two-tailed statistical tests. A difference was considered statistically significant when *P* < 0.05. The correlations between SATB1 and SATB2 expression, SATB1 and c-Myc expression or SATB2 and c-Myc expression in clinical samples were determined by Pearson correlation coefficient (r).
